# Fortification of dough with moringa, coriander, and amaranth improves the nutritional composition, health‐benefiting properties, and sensory attributes of Nigerian wheat bread

**DOI:** 10.1002/fsn3.3753

**Published:** 2023-11-06

**Authors:** P. O. Oyinloye, A. S. Ajala, N. T. Asogwa, Y. J. H. Galani

**Affiliations:** ^1^ Department of Food Science and Engineering Ladoke Akintola University of Technology Ogbomoso Nigeria; ^2^ School of Allied and Public Health Professions, Faculty of Medicine, Health and Social Care Canterbury Christ Church University Canterbury UK; ^3^ Central Research Laboratory Ilorin Nigeria; ^4^ Section of Natural and Applied Sciences, School of Psychology and Life Sciences Canterbury Christ Church University Canterbury UK

**Keywords:** amaranth, antioxidant capacity, bread, coriander, fortification, glycemic index, moringa

## Abstract

Consumption of bread can be associated with some health issues, which can be improved by fortifying it with plants that are good sources of nutrients and bioactive compounds. This study investigated the effects of fortifying bread with 3 leafy vegetables on the quality of Nigerian wheat bread. Leave powders of coriander, moringa, and amaranths were added to wheat dough at 0% (control), 1%, 3%, 5%, or 7%, and the blends obtained were used to bake vegetable breads, which were then analyzed for proximate, minerals, total phenolics, antioxidant activity, reducing sugars, glycemic index, and sensory evaluation. Results showed that vegetable fortification significantly increased bread ash (from 0.84% in control up to 1.93% in fortified bread), crude fiber (from 1.68% to 3.29%), and nutritionally important minerals Ca, Mg, P, Fe, and Zn (up to 5.2‐fold, 5.1‐fold, 18.1‐fold, 4.1‐fold, and 14.0‐fold, respectively); it reduced carbohydrates (from 65.65% down to 43.16%), crude lipids (from 2.25% down to 0.44%), and caloric value (from 1239.65 down to 1125.19 kJ/100 g), with little or no effect on proteins and moisture content. The fortification also improved the bioactive properties of the bread, as evidenced by a considerably higher phenolic content (from 0.40 up to 13.95 mg/100 g GAE) and increased antioxidant activities. There was a significant 1.1‐to 3.4‐fold decrease in the reducing sugars of composite breads with 5% and 7% vegetable powder, and the selected bread formulation with Moringa 7% lowered the glycemic index of rats by 3.5‐fold. Fortification did not generally affect the appearance and taste of the breads but decreased other sensory parameters and overall acceptability; the bread sample enriched with 1% amaranth received the highest general acceptance. In conclusion, fortifying wheat bread with the 3 vegetables improves its nutritional quality and can be recommended as a new pathway for the development of more nutritious and healthy bread.

## Graphical abstract

Three vegetables were incorporated into the dough of Nigerian wheat bread. This fortification significantly improved the nutritional profile of the bread, including an increase in ash, crude fiber, and minerals, with reduced carbohydrates, crude lipids, and caloric value. Additionally, the bread exhibited enhanced antioxidant and antimicrobial properties, which are associated with various health benefits. Moreover, the sensory evaluation of the bread showed that the addition of these vegetables did not adversely affect its overall acceptability, taste, or texture.

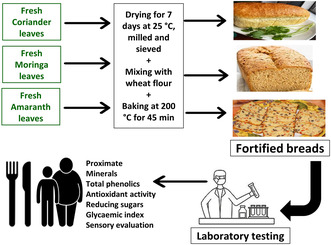

## 1 INTRODUCTION

To improve human health through nutrition, staples such as wheat bread with better nutritional value are needed. This can be achieved through the fortification of wheat flour or dough with foods rich in nutrients and bioactive compounds, such as vegetables. Bread is a staple diet consumed by people of all age groups on almost all continents (Bolarinwa et al., 2017). It is a typical baked food mostly produced with wheat flour that is rich in carbohydrates and fat but deficient in protein (12.04%–13.10%) and minerals (9.32–20.52 mg/100 g) (Ameh et al., 2013). It is a common food in most homes, eateries, and hotels and has surpassed rice as the second most popular non‐indigenous dish. Nigeria's wheat production was 90,000 t in 2021 and was far too low to satisfy the country's demand. Consequently, imported wheat flour is imported from other countries such as the US, Russia, and Ukraine to fill local flour needs for bakeries, which causes a significant outflow of foreign currency: in 2021, Nigeria imported 6.4 million ton of wheat grain and flour, representing an import value of $2.06 billion (FAO, 2023). One of the most widely eaten food items is bread, which has been readily available in Nigeria for many years and is organoleptically acceptable to people (Arunasagar et al., 2005). To improve the nutritional quality of bread, composite flours, in which wheat flour has been mixed with flour from other sources, can be used. Even though composite bread is now widely available, it still requires at least 70% wheat flour to rise properly since it contains gluten, the most abundant protein, that helps the wheat dough rise.

In the literature, the use of composite flour has been deemed successful (Olaoye et al., 2011). Fortifying bread with other agricultural products that can improve its functioning, nutritional value, and variety is a target of the country. Recently, numerous sources have emphasized the need for local crops to substitute imported wheat in bread production to reduce economic hardship (Famuwagun et al., 2016). To increase the bread's nutritional value, wheat, maize, and orange‐fleshed sweet potatoes have been added to rice bran flour, as reported by Ameh et al. (2013). Although normal (100% wheat) bread had better overall acceptability with a value of 7.95 when compared with composite bread (7.00), composite bread had significantly higher nutritional quality. However, because people have become accustomed to normal bread, manufacturers face a hurdle in reinventing composite bread and putting it in line with target consumers' expectations. In most markets, this trend is still in its early phases, but the demand is still existing, and its spread and growth are expected to continue (Awuni et al., 2017; Dhen et al., 2018; Owusu et al., 2017).

Moringa, coriander, and amaranth are low‐cost, high‐quality nutritional sources for the poor, especially in areas where malnutrition is on the rise. Vegetables' fiber content leads to a sensation of fullness and prevents constipation. Leafy vegetables are low in cost in Nigeria but high in nutritional value. Phytochemicals, protein, and minerals are abundant in vegetables, and green leafy vegetables are not only high in nutrition but also have therapeutic characteristics. On cookies, the use of vegetable powder as a food forticant has been examined (Haneen, 2015), and the results showed that minerals, fiber, and protein contents increased significantly as moringa content increased.

Consumption of bread has been associated with an increase in fat‐producing hormone (insulin), which can result in hyper‐insulinemia that can cause an increase in blood glucose. That is why there is a need to produce bread that is low in carbohydrates (Stamataki et al., 2017). Since it is practically impossible to do without eating carbohydrates such as bread in a diet, it is pertinent to investigate how the best bread can be modified by enriching it with vegetables to provide a near alternative to normal bread that can provide nourishment and yet supply the needed nutrients such as minerals, vitamins, and fiber for the betterment of human health among individuals with chronic diseases. Therefore, this work aimed to study the effect of moringa, coriander, and amaranth leaf powder on the nutritional quality of wheat bread.

## 2 MATERIALS AND METHODS

Materials used were wheat flour, dried yeast, margarine, sugar, salt, milk, egg, and water. The wheat flour was purchased from Ipata market, and the leafy vegetables, coriander, amaranth, and moringa were purchased from a local market in Ganmo, all in Kwara State, Nigeria. Fresh leaves from the vegetables were separated, de‐stalked, and washed with tap water. With a knife, the washed leafy vegetables were shredded, weighed, and dried for seven days at room temperature. The dried vegetable leaves were ground into a fine powder using a Marlex Excella dry mill (Marlex Appliances PVT, Daman) and sieved with a 176‐micron sieve to obtain a uniform powder. The powder was packaged and sealed in a polythene bag for further analysis.

### 2.1 Bread formulations

The wheat flour was sieved using a 176‐micron sieve to remove any lumps and weighed. The dough was mixed separately with each of the vegetables at varying percentage proportions (100:0, 99:1, 97:3, 95:5, and 93:7) for wheat and vegetables, respectively, as shown in Table 1. Each baking formulation experiment was made to be 200 g.

**TABLE 1:** Dough formulation of vegetable breads.

Wheat–moringa bread		Wheat–coriander bread		Wheat–amaranth bread	
Wheat (%)	Moringa (%)	Wheat (%)	Coriander (%)	Wheat (%)	Amaranth (%)
Control	0	Control	0	Control	0
99	1	99	1	99	1
97	3	97	3	97	3
95	5	95	5	95	5
93	7	93	7	93	7

### 2.2 Baking process for vegetable breads

The dough was baked using the Greene and Bovell‐Benjamin (2004) method. After weighing and mixing all the dry components, 2 g of yeast suspension in water was added. 120 mL of water was added to the mixture and further mixed to form a dough. In accordance with the adjusted bread baking criteria, the dough was kneaded, sized, sliced, and molded before being placed in greased baking pans (Famuwagun et al., 2016). The molded dough was given a 2‐h proofing period in a proofing chamber before being baked at 200°C for 45 min. The freshly baked bread samples were given 30 min to cool to room temperature, then bagged, labeled, and stored for subsequent analysis.

### 2.3 Determination of the proximate composition of bread samples

Proximate composition analysis of vegetable bread was carried out using the Association of Official Analytical Chemists [AOAC] (2012) method as follows:

#### 2.3.1 Moisture content

Two grams of bread material were added to an empty crucible after it had been weighed. It was then dried in a hot air oven for 24 h at 100°C. The crucible and its contents were cooled in a desiccator, and their weights were recorded. The moisture content was determined as a percentage of the weight loss of the sample (AOAC, 2012).

#### 2.3.2 Ash content

A bread sample (5 g) was placed into crucibles and incinerated at 550°C in a muffle furnace until light gray ash was seen and a steady weight was recorded. To prevent moisture reabsorption, the sample was cooled in a desiccator and then weighed to determine its ash content, which was expressed as a percentage of the sample weight (AOAC, 2012).

#### 2.3.3 Crude fiber

A 500‐mL conical flask was filled with 5 g (*W*_0_) of bread sample, and 100 mL of the trichloroacetic acid (TCA) digestion reagent was then added. The mixture was brought to a boil and refluxed for exactly 40 min, starting when the water first began to boil. The flask was taken out of the heater, allowed to cool for 5 min and then filtered through a 15.0 cm piece of No. 4 Whatman paper. The residue was washed with hot water and given a quick spin with a spatula before being placed in a porcelain dish. The sample was dried overnight at 105°C, and then it was moved to desiccators after drying, where it was weighed as *W*_1_. It was then burned for six hours at 500°C in a muffle furnace, allowed to cool, and weighed again as *W*_2_ (AOAC, 2012).

$$\%\text{crude fibre}=\frac{W_{1}-W_{2}}{W_{0}}\times 100$$

*W*_1_ = weight of crucible + fiber + ash; *W*_2_ = weight of crucible ash; *W*_0_ = dry weight of the food sample.

#### 2.3.4 Fat content

Two grams of bread samples were introduced in a previously weighed flat‐bottom flask and extracted using a boiling point of 40–60°C. After an 8‐h extraction, the remaining material in the flask was dried at 80°C for 30 min in an air oven to remove any leftover fat before being cooled in a desiccator after the solvent was removed by evaporation over a water bath. The flask was weighed again, and the percentage of fat was determined as a ratio of weight lost against the original sample weight (AOAC, 2012).

#### 2.3.5 Protein content

The content of crude protein was determined using the micro Kjedal method. Bread material (2 g) was digested with 25 mL of concentrated sulfuric acid and 10 g of concentrated copper and sodium sulfate (catalyst) in a 5:1 ratio. After digestion, 10 mL of the mixture and 10 mL of 40% sodium hydroxide were distilled, and the nitrogen content of the sample was determined. The protein content of the sample was obtained by multiplying its nitrogen content by 6.25 (AOAC, 2012).

#### 2.3.6 Carbohydrate content

The carbohydrate content was calculated by the difference of the remaining after all the other components (protein, lipid, ash, fiber, and water) had been measured (AOAC, 2012).

#### 2.3.7 Determination of mineral composition

Bread samples (0.5 g) were digested with 5 mL of concentrated nitric acid and 2 mL of perchloric acid. The digest was filtered and used for the determination of the mineral content of the samples (Ca, Mg, Na, P, Zn, and Fe) using a Buck 210VGS Scientific Model atomic absorption spectrophotometer (Awofolu, 2005).

### 2.4 Antioxidant analyses

#### 2.4.1 Preparation of an aqueous extract for antioxidant analyses

An aqueous extract of the vegetable bread was obtained by soaking 1 g of ground vegetable bread in 10 mL of distilled water for 24 h on a stirring plate. The sample was centrifuged at 900 rpm for 20 min, and the supernatant was collected, filtered using Whatman Filter Paper No. 589/2, and stored at 4°C for antioxidant analysis (Aderogba et al., 2005). The aqueous extract was used for total phenolic content and antioxidant assays.

#### 2.4.2 Total phenolic content

Total phenolic content was determined using the method from Makkar et al. (2009). Aqueous extract (0.1 mL) was placed in a test tube and diluted with 0.9 mL of distilled water. Then 0.5 mL of Folin–Ciocalteu reagent (1:1 with distilled water) and 2.5 mL of sodium carbonate solution (20%) were added sequentially to the test tube. The reaction mixture was quickly vortexed, the tubes were left in the dark for 40 min, and the absorbance was measured at 725 nm against the blank (distilled water in place of extract). A standard curve using gallic acid monohydrate was obtained with a linearity range of 1 to 10 μg/mL. The total phenolic content was determined using the standard curve and represented as gallic acid equivalent (GAE) in mg/100 g of extract.

#### 2.4.3 Determination of antioxidant activities

##### Hydroxyl radical‐scavenging antioxidant activity

The hydroxyl radical (OH)‐scavenging activity of the aqueous vegetable extracts was measured by the method of Kumaran and Karunakaran (2007). The hydroxyl radical was produced in a salt combination of 1.0 mL of 0.75 mM 1,10‐phenanthroline, 2.0 mL of 0.2 M sodium phosphate buffer (pH 7.4), 1.0 mL of 0.75 mM FeSO_4_, and 1.0 mL of H_2_O_2_ (0.01%, v/v). Aqueous vegetable extract (1.0 mL) was added, and the mixture was then incubated at 37°C for 30 min. The mixture's absorbance was then measured at 536 nm. The positive control was the standard vehicular carrier/medium, while the blank was deionized water. The scavenging activity was calculated by the following equation:

$$\begin{array}{c} \mathrm{OH}\;\text{Scavenging Activity}\;\left(\%\right)\\ =\frac{\text{Absorbence}\;\left(\text{Sample}\right)-\text{Absorbence}\;\left(\text{blank}\right)}{\text{Absorbence}\;\left(O\right)-\text{Absorbence}\;\left(\text{blank}\right)}\times 100 \end{array}$$

where Absorbance (O) is the absorbance of the deionized water instead of H_2_O_2_.

##### Phosphomolybdenum antioxidant activity

Phosphomolybdenum was used to measure the extracts' antioxidant activity, with ascorbic acid as the standard. Aqueous vegetable extracts (0.1 mL) were combined with 3 mL of the reagent (0.6 M sulfuric acid, 28 mM sodium phosphate, and 4 mM ammonium molybdate). The tubes were sealed and incubated for 90 min in a water bath at 95°C. After the samples had cooled to room temperature, the absorbance of each solution was measured with a spectrophotometer at 695 nm. The antioxidant capacity was measured as the ascorbic acid equivalent in mg/100 g (Makkar et al., 2009).

##### 2,2‐Diphenylpicrylhydrazyl (DPPH)‐scavenging activity

The method of Patel and Patel (2011) was followed to determine the diphenylpicrylhydrazyl (DPPH)‐scavenging activity. Four milliliters of a 0.1 mmol/L methanolic solution of DPPH were mixed with 0.2 mL of extract or 0.2 mL of distilled water for the blank. Utilizing a spectrophotometer, the absorbance at 517 nm was measured in comparison to the prepared blank after 30 min of incubation at room temperature and in the dark. The DPPH‐scavenging properties of the breads were compared with the scavenging properties of butylated hydroxyl toluene used as a standard, and determined in μg/mL.

### 2.5 Reducing sugar assay

Reducing sugar was assayed in solutions using the 3,5‐dinitrosalicylic acid (DNS) method described by Saqib and Whitney (2011). Sugar solutions (1 mL) from a series of standard D‐glucose solutions of 0.1, 0.2, 0.3, 0.4, 0.5, 0.6, and 0.7 mg/mL concentrations were added to screw‐cap tubes containing 4 mL of a 0.02 mg/mL prepared standard DNS reagent. The standard DNS was prepared by adding 1 g of 3,5 DNS, 1 g of NaOH, 200 mg of phenol crystal, 50 mg of sodium sulfite, and 40 mg of potassium sodium tartrate to 100 mL of water sulfite and mixing well on a magnetic stirrer. The tubes were heated to a boil for 5 min, then quickly cooled with crushed ice before being returned to a water bath to reach room temperature. Absorbance was measured at 540 nm, using a spectrophotometer (UV‐1601, Japan). The experiments were repeated at 1, 2, and 3 h for in vitro digested vegetable breads and controls. A standard curve was used to determine the reducing sugar concentration in relation to each absorbance value.

### 2.6 Glycemic index determination

Healthy Wistar rats were randomly grouped into two groups: one group was fed the composite bread (Moringa 7%), while the other (control) was fed the bread without vegetables. The blood glucose of the rats was checked by a needle prick to the tip of the tail. The tail was gently massaged from the base upwards to generate blood droplets. The droplet of blood was placed on a glucose strip inside the glucometer to ascertain that they were not diabetic. The method for measuring and determining the glycemic index (GI) of the breads followed the recommendations of the Food Fortification Initiative (FFI, 2014). The rats attended each testing session after a 10 h overnight fast. Portion sizes of 50 g of accessible carbohydrates were found to be too large for individuals to ingest comfortably within 15 min for some of the breads. As a result, portions analyzed delivered 25 g of accessible carbohydrates, with a 25 g glucose reference made up of 125 mL water as a comparison. Portions of two breads containing 50 g of accessible carbohydrates were also examined to compare the GI values and determine whether the different portion sizes gave the same result. In the fasting state (0 min) and 15, 30, 45, 60, 90, and 120 min following the start of food consumption, blood glucose levels were determined in capillary whole blood taken by a tail prick.

### 2.7 Sensory evaluation

The five bread samples were coded and presented to a panel of fifty semi‐trained panelists for sensory evaluation. The bread's color, flavor, taste, texture, and general acceptability were evaluated on a Hedonic scale with 9 points, where 9 represents extreme like and 1 represents extreme dislike (Ajala et al., 2018).

### 2.8 Statistical analysis

An analysis of variance was used to compare the results of the chemical tests and the sensory rating using SPSS (20.0 version), and means were separated using Duncan's multiple range test at a 95% confidence level. The analysis of data was conducted (for the experimental animals) with Instat Statistical software, and the experiment was done in triplicate. Comparisons among groups using one‐way ANOVA and post hoc analyses were done with the Tukey test for pair‐wise comparison at a 95% confidence level.

## 3 RESULTS AND DISCUSSION

### 3.1 Proximate composition

The results of the proximate composition of the formulated vegetable bread and the control sample are presented in Table 2. According to Ojo et al. (2021) and Brun et al. (2013), the moisture content of food is an essential indicator of vulnerability to microbial spoilage. Furthermore, increased moisture content increases the growth of microbes, making food more susceptible to degradation and extending its perishability (‌Temple et al., 1996). The values of moisture ranged from 25.72% to 30.05%, while the range of values for ash, carbohydrate, protein, lipid, and fiber were 0.78%–1.93%, 43.16%–65.00%, 1.20%–3.91%, 0.44%–2.27%, and 1.68%–3.29%, respectively. The values were significantly different within the same vegetable group sample as well as with different vegetable group samples, as shown in the Table. The results obtained agreed with those of Bibiana et al. (2014). There was a significant difference in moisture contents in the formulated vegetable bread samples when compared with the control (26.08%), and the value of the moisture content was not dose‐dependent. The low moisture content observed with some of the fortified breads in this study, therefore, indicates low potential growth of bacteria and fungi in the composite breads.

**TABLE 2:** Proximate content of formulated vegetable breads.

Bread sample	Moisture %	Ash %	CHO %	Protein %	Crude lipid %	Crude fiber %	Caloric value kJ/100 g
Coriander 1%	25.91 ± 2.38^a^	1.03 ± 0.08^a^	57.81 ± 1.40^a^	3.29 ± 0.05^a^	1.34 ± 0.81^a^	1.82 ± 0.34^a^	1216.17 ± 6.13^b^
Coriander 3%	28.13 ± 0.73^d^	1.23 ± 0.05^a^	54.23 ± 3.28^a^	3.27 ± 0.05^a^	1.34 ± 0.59^a^	1.84 ± 0.14^a^	1177.53 ± 1.82^a^
Coriander 5%	26.81 ± 1.35^b^	1.45 ± 0.05^a^	52.73 ± 0.69^a^	3.19 ± 0.77^a^	1.27 ± 0.01^a^	1.85 ± 0.07^a^	1150.32 ± 24.60^a^
Coriander 7%	29.19 ± 4.17^e^	1.93 ± 0.03^a^	43.16 ± 2.01^a^	2.65 ± 0.59^a^	0.44 ± 0.08^b^	2.07 ± 0.16^a^	1138.95 ± 4.13^a^
Moringa 1%	27.47 ± 0.33^c^	0.78 ± 0.19^b^	56.61 ± 0.98^a^	3.16 ± 0.67^a^	2.27 ± 0.04^a^	2. 06 ± 0.36^a^	1255.41 ± 6.43^c^
Moringa 3%	26.25 ± 0.32^ab^	0.84 ± 0.12^b^	54.24 ± 0.22^a^	2.72 ± 0.77^a^	1.88 ± 0.87^b^	2.15 ± 0.33^a^	1205.35 ± 16.24^ab^
Moringa 5%	28.28 ± 1.56^d^	1.03 ± 0.12^a^	51.55 ± 1.54^a^	2.17 ± 0.69^a^	1.53 ± 0.16^a^	2.40 ± 0.67^a^	1165.94 ± 20.12^a^
Moringa 7%	27.28 ± 2.56^c^	1.23 ± 0.17^a^	47.76 ± 2.80^a^	1.20 ± 0.08^b^	1.05 ± 0.78^a^	3.29 ± 0.23^b^	1125.19 ± 16.31^a^
Amaranth 1%	26.24 ± 3.89^ab^	0.84 ± 0.00^b^	65.53 ± 1.68^b^	3.36 ± 0.05 ^a^	2.15 ± 0.13^a^	1.73 ± 0.13^a^	1205.51 ± 9.78^ab^
Amaranth 3%	30.05 ± 4.04^f^	0.87 ± 0.10^b^	65.15 ± 5.37^b^	3.02 ± 0.90^a^	1.96 ± 0.39^a^	1.86 ± 0.72^ab^	1141.26 ± 19.32^a^
Amaranth 5%	25.72 ± 2.64^a^	1.46 ± 0.07^a^	61.48 ± 6.49^b^	3.91 ± 0.27^a^	1.07 ± 0.34^a^	2.26 ± 0.38^ab^	1332.73 ± 16.66^d^
Amaranth 7%	26.06 ± 0.01^a^	1.53 ± 0.02^a^	54.53 ± 1.85^a^	3.74 ± 0.90^a^	0.69 ± 0.08^a^	2.53 ± 0.15^ab^	1196.89 ± 5.54^a^
Control	26.08 ± 0.09^a^	0.84 ± 0.06^b^	65.65 ± 0.56^b^	3.50 ± 0.66^a^	2.25 ± 0.28^a^	1.68 ± 0.20^a^	1239.65 ± 18.81^bc^

*Note*: Data are presented as mean ± standard deviation; values with the same alphabetic superscript within a column are not significantly different (*p* < .05).

The ash contents of the formulated vegetable bread samples ranged from 1.03%–1.93% for coriander, 0.78%–1.23% for moringa, and 0.84%–1.54% for amaranth. A significant dose‐dependent increase was observed within the same vegetable group sample as well as with different vegetable group samples; there were no significant differences except for moringa samples. Among all the vegetables used, the concentrations of 5% and 7% were significantly different from the control bread. This indicates that the vegetable could be a source of minerals and confirms the high mineral content of the prepared samples.

The main goal of food modification for people with chronic diseases such as diabetes, hypertension, cardiovascular diseases, and obesity is to reduce their intake of causative agents such as glucose and saturated fat (Ludwig et al., 2018). The carbohydrate content of all the vegetable breads was significantly lower compared to the carbohydrate content of the control (65.65 ± 0.56%) except for amaranth 1% and 3%, which were not significantly different. The observed reduction in carbohydrates may be due to the inclusion of vegetables.

Proteins may be advantageous for young and healthy people with high levels of anabolism compared to catabolism, as they are used by the body to build and repair tissues, as well as to produce enzymes, hormones, and other substances. However, for diabetic patients, high protein metabolism puts a lot of strain on the kidneys, which are already prone to diabetic nephropathy. There was a reduction in protein content in all the composite breads compared with the control. However, there was no significant difference between the composite breads and the control, except for Moringa 7%. The result from Table 2 therefore signifies that wheat is the major source of protein in bread because there is an important reservoir of gluten in wheat grain. Protein is an essential component of every cell in the human body; the formulated vegetable breads, therefore, demonstrate to be a good nutraceutical formulation for individuals who are expected to cut down on protein intake (Helal et al., 2019).

Although lipid supply is needed for calorie and transport vehicles for fat‐soluble vitamins, excess fat in the diet has been shown to alter overall nutrient intake and may exacerbate micronutrient deficiencies in vulnerable populations (PAHO/WHO, 2003). Consuming too much fat, especially saturated fat, increases the risk of obesity and cardiovascular disease (Milner & Allison, 1999). The crude lipid observed from all the vegetable‐formulated breads ranged from 0.44%–1.34% with no significant difference for coriander, 1.05%–2.27% for moringa with no significant difference except the sample with 3%, and 0.69%–2.15% for amaranth with no significant difference. A significant reduction in crude lipid was observed with a concomitant increase in the percentage of vegetables used, particularly in the higher doses. An explanation for these findings may also be due to the bulk weight of the vegetable, which was also observed to be dose‐dependent (Table 2). Also, the amount of lipid from the vegetables may not have added any significant concentration value to the lipid, but it would have enriched the quality of the fat used since the fat used for baking the bread, which is margarine (saturated fat), would have been supplemented with unsaturated fat, which is typically present in plant lipid. The use of essential fatty acids has been found to help avoid coronary heart disease, hypertension, type 2 diabetes, and kidney diseases (Khan et al., 2008). The low lipid content of the formulated vegetable bread may be necessary to prolong its shelf life because excess fats are known to undergo lipid peroxidation and may result in food spoilage.

The crude fiber contents observed in Table 2 show that the formulated bread fiber increased with an increase in vegetable fortification, which suggests that the vegetables, being a fiber‐rich source, contributed to the fiber contents of the formulated bread. There was no significant difference within the group of coriander breads, nor within the groups of amaranth bread and the group of moringa bread except for the sample 7% inclusion. High‐fiber food slows the absorption of sugar and improves blood sugar levels. They also tend to be more filling; hence, one may likely eat less and stay satisfied longer. Also, they increase the weight of stool and soften it, thereby decreasing the chances of constipation, which is a common occurrence among diabetics (Gill et al., 2021).

Calorie as an energy source provides materials for body growth and repair as well as energy for all bodily functions, including breathing, digesting food, and remaining warm. They also support the immune system (Chauhan et al., 2018). The calorific values of bread samples ranged from 1125.19 to 1332.73 kJ and were significantly different from one another both within and among the group of vegetable bread samples, with the lowest value found in moringa 7% bread and the highest value found in amaranth 5% bread. The energy content of the bread samples in this study was comparable to the values reported by Refat et al. (2020). From these values, it implies that vegetable bread is capable of providing enough energy to satisfy the needs of consumers. These nutrients provide materials for body growth and repair as well as energy for all bodily functions, including breathing, digesting food, and remaining warm. They also support the immune system (Chauhan et al., 2018).

### 3.2 Mineral content

Minerals are important micronutrients for the good functioning of the body, and adequate amounts are needed from the diet. Besides, human activities such as industrial and consumer waste cause heavy metals to build up in the water supply, which can then contaminate foods and pose health problems to consumers. The mineral composition of the vegetable bread is shown in Table 3. Arsenic was not detected in all the concentrations of the formulated bread as well as the control. Also, except for samples of bread with 5% moringa and 7% moringa, lead was not detected in all the formulated bread and the control group used in this research. Both arsenic and lead are classified as heavy metals, according to the WHO (1998). Because of the acceptable lead content, moringa‐enhanced bread can be considered safe for consumption. The amounts of lead found in samples of bread with 5% moringa and samples of bread with 7% are within the provisional tolerable weekly intake (PTWI) of total lead, which is 5 ppm, as prescribed by the Food and Agriculture Organization/World Health Organization Joint Expert Committee on Food Additives (WHO/FAO‐JECFA, 2003). The presence of lead and arsenic may have been because of contamination from the plant source using pesticides or herbicides (Nema et al., 2016). This finding corroborates the results reported by Annan et al. (2010) and further highlights the need to screen for heavy metals when using plant‐base additives in nutritional formulation.

**TABLE 3:** Mineral content of the formulated vegetable breads.

Bread sample	Arsenic (μg/g)	Lead (μg/g)	Zinc (μg/g)	Iron (μg/g)	Phosphorus (μg/g)	Calcium (μg/g)	Magnesium (μg/g)
Coriander 1%	ND	ND	2.86 ± 0.01^b^	0.62 ± 0.01 ^a^	0.94 ± 0.03^a^	4.42 ± 0.49^a^	2.56 ± 0.89^ab^
Coriander 3%	ND	ND	2.91 ± 0.02^b^	0.70 ± 0.02 ^a^	1.07 ± 0.10^b^	15.73 ± 0.25^b^	3.78 ± 0.43^c^
Coriander 5%	ND	ND	3.04 ± 0.07^b^	0.78 ± 0.05 ^a^	3.25 ± 0.19^c^	18.85 ± 0.15^b^	4.21 ± 0.22^cd^
Coriander 7%	ND	ND	3.91 ± 0.07^bc^	0.82 ± 0.01 ^a^	5.94 ± 0.30^d^	25.24 ± 0.15^c^	4.78 ± 0.56 ^cd^
Moringa 1%	ND	ND	3.07 ± 0.07^b^	0.81 ± 0.02 ^a^	3.91 ± 0.29^c^	10.14 ± 0.05^b^	2.34 ± 0.12^ab^
Moringa 3%	ND	ND	3.54 ± 0.36^b^	0.85 ± 0.02 ^a^	5.26 ± 1.10^d^	13.33 ± 0.10^b^	4.55 ± 0.36^cd^
Moringa 5%	ND	0.89 ± 0.01^a^	5.30 ± 0.46^d^	1.21 ± 0.01^b^	5.79 ± 0.63^d^	20.96 ± 0.25^bc^	6.34 ± 0.45^d^
Moringa 7%	ND	0.76 ± 0.05^a^	5.46 ± 0.62^d^	2.98 ± 0.24^c^	8.70 ± 0.32^e^	25.24 ± 0.05^c^	6.89 ± 0.33^d^
Amaranth 1%	ND	ND	3.10 ± 0.15^b^	0.80 ± 0.01 ^a^	1.63 ± 0.08^b^	4.36 ± 0.05^a^	3.32 ± 0.21^c^
Amaranth 3%	ND	ND	3.40 ± 0.29^b^	0.83 ± 0.04^a^	3.59 ± 0.18^c^	6.55 ± 0.64^a^	3.13 ± 1.05^c^
Amaranth 5%	ND	ND	3.89 ± 0.21^bc^	0.82 ± 0.81^a^	5.57 ± 0.32^d^	19.13 ± 0.15^b^	4.05 ± 0.88^d^
Amaranth 7%	ND	ND	4.03 ± 0.24^c^	0.80 ± 0.93 ^a^	11.41 ± 0.78^f^	20.83 ± 0.15^bc^	8.45 ± 2.12^e^
Control	ND	ND	0.39 ± 0.00^a^	0.73 ± 0.02^a^	0.63 ± 0.00^a^	4.90 ± 0.10^a^	1.67 ± 0.67^a^

*Note*: Data are presented as mean ± standard deviation; values with the same alphabetic superscript within a column are not significantly different (*p* < .05).

Abbreviation: ND, not detected.

The contents of zinc, phosphorus, calcium, and magnesium except iron in all the formulated bread were all significantly different when compared with the control. They also showed a dose‐dependent composition of the mineral content. The concentration of zinc ranged from 2.86–3.91 ppm for coriander, 3.07–5.46 ppm for moringa, and 3.10–4.03 ppm for amaranth, while the non‐fortified formulation had 0.39 ppm. Phosphorus concentrations in the formulation ranged from 0.94–5.94 ppm for coriander, 0.391–8.70 ppm for moringa, and 1.63–11.41 ppm for amaranth. Calcium had a concentration range of 4.42–25.24 ppm, 10.14–25.24 ppm, and 4.36–20 ppm for coriander, moringa, and amaranth breads, respectively. The values of the mineral content of breads obtained in this study are on par with those reported by Ojo et al. (2021).

The recommended daily intake (RDI) of zinc is 15 mg/day, iron is 15 mg/day, phosphorus is 1000 mg/day, and calcium is 1000 mg/day for young adults, as reported by Kim and Choi (2013). All the formulated vegetable breads used in this research were richer in these minerals than the control bread sample. However, this is often balanced by other sources of nutrients that accompany bread while eating to meet the RDI (Amnah, 2018). Microelements rose in all formulations as vegetable fortification increased, implying that the vegetables enriched the main flour in the designed bread with minerals. This is of great importance from a nutritional and health point of view since iron, zinc, and calcium are among the most common micronutrient deficiencies, especially in sub‐Saharan Africa. Iron deficiency leads to anemia (which results in a decrease in the oxygen‐carrying capacity of blood through the body), as well as impaired immune and endocrine function. Zinc acts as a stabilizer protects membrane structure and cell components, and is responsible for the efficient functioning of the enzyme system. Adequate zinc intake is necessary for optimal immune function. Calcium, the key mineral in teeth and bone formation, can reduce the risk of fractures, osteoporosis, and diabetes in some populations, and its supplementation during pregnancy helps to prevent many gestational disorders (Galani et al., 2020).

### 3.3 Antioxidant properties and total phenolic content

Aerobic organisms produce free radicals as part of their normal metabolic process. These radicals are highly reactive; they can oxidate proteins, lipids, and DNA, causing oxidative damage as a result. Antioxidants serve a critical function in protecting the human body from free radicals (Martemucci et al., 2022). Food exerts its antioxidant characteristics through a variety of methods, including free radical scavenging activities and reducing characteristics. The bread samples were evaluated for total phenolic content and antioxidant activity, and the results are presented in Table 4. The bread's free radical hydroxyl scavenging activities were concentration‐dependent; all the fortified bread showed significantly higher activities than the control sample (53.08%), and the highest value (84.54%) was obtained with the coriander 7% formulation. However, the samples were significantly different within the vegetable types and within vegetable concentrations. A similar concentration‐dependent pattern was observed with phosphomolybdenum antioxidant activity, with the highest value of 205 mg/100 g from 7% moringa samples and the lowest value of 17 mg/100 g from the control sample. The samples exhibited significant differences among and within the vegetable groups, except amaranth samples. The values of DPPH‐scavenging activity varied from the lowest value of 509 μg/mL in the control sample to the highest value of 772 μg/mL in the 7% amaranth sample. There was no significant difference within the vegetable groups, but they all differed from the control. This demonstrates that the incorporation of vegetables (coriander, moringa, and amaranth) in wheat flour for bread formulation contributed to the increased antioxidant activity of the bread.

**TABLE 4:** Total phenolic content and antioxidant properties of vegetable breads.

Bread sample	Hydroxyl radical‐scavenging capacity (%)	Phosphomolybdenum antioxidant activity (mg/100 g)	DPPH‐scavenging activity (μg/mL)	Total phenolic content (mg/100 g GAE)
Coriander 1%	76.08 ± 0.56^b^	164.34 ± 0.00^b^	670.90 ± 0.70^b^	11.90 ± 1.06^a^
Coriander 3%	79.72 ± 0.28^b^	167.13 ± 0.14^b^	687.20 ± 0.40^b^	12.255 ± 0.05^a^
Coriander 5%	83.22 ± 3.78^c^	185.73 ± 0.35^c^	693.10 ± 9.30^b^	13.30 ± 0.00^a^
Coriander 7%	84.54 ± 0.21^c^	199.86 ± 0.21^c^	706.65 ± 0.45^b^	13.90 ± 0.10^a^
Moringa 1%	58.39 ± 1.05^a^	168.81 ± 0.14^b^	701.65 ± 0.45^b^	11.95 ± 0.05^a^
Moringa 3%	65.10 ± 0.50^a^	173.29 ± 0.14^bc^	717.40 ± 0.10^b^	12.30 ± 0.00^a^
Moringa 5%	72.10 ± 0.07^ab^	185.31 ± 0.28^c^	720.55 ± 0.15^b^	12.50 ± 0.00^a^
Moringa 7%	92.10 ± 1.05^d^	204.76 ± 0.00^d^	730.35 ± 0.35^b^	13.30 ± 0.00^a^
Amaranth 1%	59.31 ± 0.14^a^	126.71 ± 0.21^b^	711.35 ± 0.95^b^	12.05 ± 0.05^a^
Amaranth 3%	63.56 ± 0.28^a^	154.48 ± 0.14^b^	722.60 ± 0.30^b^	12.65 ± 0.05^a^
Amaranth 5%	79.85 ± 0.60^b^	162.25 ± 0.21^b^	731.60 ± 2.40^b^	13.20 ± 0.00^a^
Amaranth 7%	80.86 ± 0.00^c^	165.94 ± 0.14^b^	772.40 ± 0.80^b^	13.95 ± 0.15^a^
Control	53.08 ± 6.20^a^	17.10 ± 0.02^a^	508.8 ± 28.30^a^	0.40 ± 0.10^b^

*Note*: Data are presented as mean ± standard deviation. Values with the same alphabetic superscript within a column are not significantly different (*p* < .05).

The total phenolic contents in the bread samples varied from 0.40–13.95 mg/100 g GAE and were not significantly different within the vegetable groups, but they were all higher than the control. All the vegetable‐fortified breads showed significantly higher (10‐fold on average) phenolics than the control; the highest was found in the sample with 7% amaranth bread. Furthermore, the phenolic value in the bread sample was concentration‐dependent, indicating the presence of phenolic components, such as tannins and flavonoids, in the vegetables, which are known to have antioxidant properties (Aderogba et al., 2005). It was also observed from the results that the amount of phenolics in each formulation follows the same pattern as OH radical scavenging activity as well as TAC values. Previous authors have demonstrated a relationship between the total antioxidant capacity and total phenolic content of some fruits; for instance, Javanmardi et al. (2003) reported that of *Adina cordifolia*; Galani, Patel, et al. (2017) reported those of amaranth, dill, onion, fenugreek, and spinach; and Galani, Mankad, et al. (2017) reported the antioxidant activities of eleven potato varieties (*Solanum tuberosum* L.).

### 3.4 Reducing sugar content

The reducing sugar content for the formulated vegetable and control breads is presented in Table 5. The reducing sugar ranged from 35.07–109.42 mg/100 g for coriander, 91.56–122.81 mg/100 g for moringa, and 63.7–104.90 mg/100 g for amaranth, while the control bread had a value of 120.39 mg/100 g. Significant differences were observed among the different vegetables and among the different concentrations. The reducing sugars decrease with an increase in the concentration of the vegetables, and this could be due to the bulk weight resulting from the fiber content of the vegetables, which assimilated the sugar content as earlier reported under the proximate result in this study and supported by Gill et al. (2021) that vegetables contain high fiber content, which could absorb some excess nutrients such as sugar in the body and purge away from the bowel as stool. The bread formulated with 7% moringa had the lowest reducing sugar; therefore, it was used further to determine the GI in the test animals.

**TABLE 5:** Reducing the sugar content of the vegetable breads.

Bread sample	Reducing sugar content (mg/100 g)
Coriander 1%	122.81 ± 0.56^e^
Coriander 3%	120.69 ± 0.13^e^
Coriander 5%	95.52 ± 0.65^c^
Coriander 7%	91.56 ± 0.32^c^
Moringa 1%	109.42 ± 0.28^d^
Moringa 3%	91.88 ± 1.27^c^
Moringa 5%	62.96 ± 0.08^b^
Moringa 7%	35.07 ± 0.07^a^
Amaranth 1%	104.9 ± 1.29^d^
Amaranth 3%	98.03 ± 0.21^c^
Amaranth 5%	91.16 ± 0.45^c^
Amaranth 7%	63.7 ± 1.57^b^
Control	120.39 ± 0.70^e^

*Note*: Data are presented as mean ± standard deviation; values with the same alphabetic superscript within a column are not significantly different (*p* < .05).

### 3.5 Glycemic index (GI)

The GI of the 7% moringa‐formulated vegetable bread and control were 3.87% and 11.11%, respectively, and were significantly different, as shown in Table 6. These values were lower than those reported by Xavier (2008), which ranged from 55% to 89% for white bread. Low‐GI foods have a value of less than 55%, intermediate‐GI meals have a value of more than 55% but less than 70%, and high‐GI foods have a value of more than 70% (Barrett & Udani, 2011). Moringa bread showed a 3.5‐fold reduction in GI when compared with control bread. This may be attributed to the high fiber, low carbohydrate, and low reducing sugar content of the moringa‐formulated vegetable bread. Therefore, moringa bread can be considered a low‐glycemic food (Barrett & Udani, 2011), while the control (non‐fortified) bread is a high‐glycemic snack. According to Mlotha et al. (2015), low‐GI foods have a slower, more gradual glucose release, which leads to more normal postprandial blood glucose levels. In the first 2 h following a meal, meals with a high GI produce a higher postprandial blood glucose peak and a greater total blood glucose response than those with a low GI. Resistant starches, which serve as dietary fiber and could slow down digestion in the small intestine, are included in foods to lower the GI of a meal (Englyst & Englyst, 2005). These resistant starches have been identified in seeds, legumes, and vegetables, and the raw materials employed in this investigation all fall into this group. Oboh et al. (2010) equally reported that pigeon pea seeds possessed a low GI. The consumption of foods with a low GI is linked to improved health. As a result, the composite vegetable bread could be used as a functional dietary snack for people who need to eat foods with a low GI.

**TABLE 6:** Glycemic index of 7% moringa‐formulated vegetable bread.

Sample	Weight of animals (g)	Blood glucose (mmol/L) 0 min	Blood glucose (mmol/L) 30 min	Blood glucose (mmol/L) 60 min	Glycemic index (%)	Glycemic load
Test (Moringa 7%)	200.33 ± 44.19^a^	6.43 ± 0.40^a^	6.47 ± 0.59^a^	7.33 ± 0.29^a^	3.87 ± 0.59^a^	23.12 ± 0.25^a^
Control	239.00 ± 24.56^a^	7.13 ± 2.70^b^	8.23 ± 3.44^b^	8.20 ± 2.78^b^	11.11 ± 2.05^b^	50.93 ± 9.79^b^

*Note*: Data are presented as mean ± standard deviation. Values with the same superscript within a column are not significantly different (*p* < .05).

### 3.6 Sensory analysis

The results of the sensory analysis of the formulated breads are presented in Table 7. It is observed that there was no significant difference in the appearance except for the control and bread containing 7% of moringa vegetables. Likewise, there was no significant difference in taste except in moringa, with 7% inclusion. However, there was a significant difference in flavor, texture, color, aroma/smell, crumb smoothness, mouthfeel, and overall acceptability of all the several concentrations used for the formulations by the panelists' ratings compared to the control. It appears from the table that the vegetable inclusion in the bread increased the aroma and smell of the bread, which attracted the panelists. For instance, there were no significant differences between the vegetable breads in the control sample, especially with 1%, 5%, and 7% coriander samples, 3% and 7% moringa samples, and 3%, 5%, and 7% amaranth samples. Hence, it can be deduced that the inclusion of these vegetables in the bread within these ranges would not significantly affect its smell and aroma. Also, the values of bread crumb smoothness show that the control sample had the best smoothness with a hedonic value of 8 when compared with vegetable bread samples.

**TABLE 7:** Sensory evaluation of the formulated vegetable breads.

Sample	Appearance	Taste	Flavor	Texture	Color	Aroma/smell	Crumb smoothness	Mouthfeel	Overall acceptability
Coriander 1%	7.10 ± 1.93^b^	7.52 ± 1.53^b^	7.52 ± 1.38^b^	7.69 ± 1.23^b^	7.07 ± 1.34^b^	7.21 ± 1.84^a^	7.45 ± 1.24^b^	7.86 ± 1.27^b^	7.62 ± 1.24^b^
Coriander 3%	7.72 ± 1.07^b^	7.79 ± 1.05^b^	7.76 ± 1.02^b^	7.69 ± 1.14^b^	7.72 ± 0.92^b^	7.83 ± 0.89^b^	7.76 ± 1.09^b^	7.97 ± 0.98^b^	7.79 ± 0.98^bc^
Coriander 5%	7.14 ± 1.66^b^	7.24 ± 1.27^b^	6.90 ± 1.45^a^	7.07 ± 1.39^a^	6.90 ± 1.50^a^	7.07 ± 1.51^a^	7.24 ± 1.33^b^	7.62 ± 1.35^b^	7.76 ± 0.99^b^
Coriander 7%	6.97 ± 1.76^a^	6.97 ± 1.57^a^	6.69 ± 1.71^a^	7.03 ± 1.21^a^	6.72 ± 1.91^a^	6.97 ± 1.96^a^	6.76 ± 1.85^a^	7.07 ± 1.65^a^	6.83 ± 1.65^a^
Moringa 1%	7.72 ± 1.71^b^	7.45 ± 1.76^b^	7.79 ± 1.17^b^	7.79 ± 1.57^b^	7.28 ± 1.39^b^	7.57 ± 1.77^ab^	7.52 ± 1.78^b^	7.61 ± 1.55^b^	7.55 ± 1.45^b^
Moringa 3%	7.89 ± 1.29^b^	7.68 ± 1.36^b^	7.54 ± 1.37^b^	7.50 ± 1.43^b^	7.79 ± 1.34^b^	7.39 ± 1.13^a^	7.64 ± 1.42^b^	7.71 ± 1.49^b^	7.68 ± 1.22^b^
Moringa 5%	7.57 ± 1.23^b^	7.36 ± 1.19^b^	7.21 ± 1.26^b^	7.46 ± 1.17^ab^	7.21 ± 1.50^b^	7.50 ± 1.48^ab^	7.64 ± 1.22^b^	7.54 ± 1.23^b^	7.61 ± 1.13^b^
Moringa 7%	6.57 ± 1.75^a^	6.50 ± 1.86^a^	6.56 ± 1.95^a^	7.25 ± 1.27^a^	6.32 ± 1.66^a^	6.50 ± 2.17^b^	6.86 ± 1.43^a^	6.82 ± 1.68^a^	6.68 ± 1.59^a^
Amaranth 1%	8.32 ± 0.86^c^	7.93 ± 0.81^b^	7.89 ± 0.99^b^	7.54 ± 0.99^b^	8.07 ± 0.98^c^	8.00 ± 1.09^b^	7.96 ± 0.99^b^	7.82 ± 0.95^b^	7.82 ± 0.95^bc^
Amaranth 3%	7.29 ± 1.41^b^	7.29 ± 1.41^b^	7.29 ± 1.41^b^	7.29 ± 1.41^a^	7.29 ± 1.41^b^	7.29 ± 1.41^a^	7.29 ± 1.41^b^	7.29 ± 1.41^b^	7.29 ± 1.41^b^
Amaranth 5%	7.50 ± 1.26^b^	7.25 ± 1.18^b^	7.21 ± 1.57^b^	7.39 ± 1.23^a^	7.14 ± 1.43^b^	7.25 ± 1.43^a^	7.11 ± 1.37^b^	7.04 ± 1.43^a^	7.04 ± 1.37^a^
Amaranth 7%	7.11 ± 2.13^b^	7.04 ± 1.62^b^	6.89 ± 1.57^a^	7.00 ± 1.70^a^	6.82 ± 1.68^a^	7.00 ± 1.74^a^	6.86 ± 1.58^a^	6.96 ± 1.67^a^	6.86 ± 1.69^a^
Control	8.15 ± 0.92^c^	7.85 ± 1.05^b^	7.81 ± 1.02^b^	7.88 ± 1.14^b^	7.85 ± 1.16^b^	7.77 ± 1.14^a^	8.00 ± 0.98^c^	7.92 ± 1.20^b^	8.23 ± 1.07^c^

*Note*: Data are presented as mean ± standard deviation. Values with alphabetic superscript^a^ within a column are significantly different (*p* < .05) compared with the control. Values with different numerical superscripts on different doses within a vegetable species are significantly different (*p* < .05).

Factors that may affect the color composition of bread with 7% moringa, inclusion may include the proximate and mineral composition of moringa particularly the high iron content (recall Table 3), which undergoes oxidation and darkens the leaf color during drying resulting from drying temperature and duration. It was observed that the color rating of the moringa sample with 7% inclusion also affected its appearance and overall acceptability rating. Color is an important organoleptic feature that increases product acceptance; hence, low color ratings of any food can reduce acceptability (Aduni et al., 2016). The color rating of the other assessed samples was within acceptable ranges, making them appealing to panelists. However, the highest concentrations of the vegetables used, namely coriander, moringa, and amaranth, with 7% inclusion, respectively, in general, had the lowest rating score in their groups.

The coriander sample with the highest inclusion had the best rating in taste, followed by the control sample, and then closely followed by coriander with 5% fortification. However, the taste ratings of all the samples were within an acceptable range. The best score rating of the amaranth sample with the lowest inclusion would be because of the better tasting quality of the amaranth leaf. Amaranths are grown principally for vegetable use and can be eaten without prolonged processing. Because of the taste of amaranth, flavor additives may not be required to be added to the vegetable bread to increase the taste ratings any further. Artificial flavors have recently been chastised by customers due to the risk of heavy metal contamination; as a result, they consider items fortified with artificial flavors to be unhealthy (FFI, 2014).

The control sample (7.88) had the best texture rating and was followed by the 1% moringa bread sample (7.79), while bread with 7% amaranth received the least consistent rating of 7.00. The texture parameters in this investigation are very similar to those reported by Ajala et al. (2018) for bread manufactured using plantains. The consistency of the flour determines the texture of the bread. A very hard texture would require increased effort to chew and swallow (Kulkarni et al., 1990) and therefore may limit consumers' acceptability.

## 4 CONCLUSION

This research investigated the influence of the addition of coriander, moringa, and amaranth on the quality attributes of wheat bread. The fortification of bread with those vegetables was found to improve the nutritional quality and organoleptic characteristics of the breads but significantly decrease their general acceptability. The modified vegetable breads at the high concentrations of 5% and 7% vegetable powder used in this research showed a significant decrease in reducing sugar. Moringa bread, having shown the most significant decrease in reducing sugar among all vegetables used in this study, was found to have a 3.5‐fold lower GI when compared with the control. This makes bread fortified with 7% moringa to be classified as low‐GI food, which is suitable for a diabetic diet.

## AUTHOR CONTRIBUTIONS

**P. O. Oyinloye:** Data curation (equal); formal analysis (lead); methodology (supporting); validation (equal); visualization (equal); writing – original draft (lead). **A. S. Ajala:** Conceptualization (lead); data curation (lead); formal analysis (supporting); investigation (lead); methodology (equal); supervision (lead); writing – original draft (supporting); writing – review and editing (supporting). **N. T. Asogwa:** Conceptualization (equal); investigation (equal); methodology (supporting); writing – review and editing (supporting). **Y. J. H. Galani:** Data curation (equal); methodology (equal); validation (equal); visualization (equal); writing – original draft (supporting); writing – review and editing (lead).

## FUNDING INFORMATION

This research was not funded.

## CONFLICT OF INTEREST STATEMENT

The authors declare no conflict of interest.

## Data Availability

The data that support the findings of this study are available in the article.
